# Quality and Readability of ChatGPT Performance on Parkinson's Disease

**DOI:** 10.1002/mdc3.70700

**Published:** 2026-06-06

**Authors:** Gabriele Riccio, Rosa Coppola, Maria Rosaria Chiariello, Augusta Giglio, Anna De Rosa

**Affiliations:** ^1^ Department of Neurosciences and Reproductive and Odontostomatological Sciences Federico II University Naples Italy

**Keywords:** artificial intelligence, ChatGPT, medical information, Parkinson's disease

## Abstract

**Background:**

Chat Generative Pre‐trained Transformer (ChatGPT) is increasingly used for medical education and healthcare counseling.

**Objective:**

To evaluate the reliability, quality, usefulness, and readability of ChatGPT‐4o responses to professional medical quizzes and patient frequently asked questions (FAQs) about Parkinson's disease (PD).

**Methods:**

Twenty‐two quizzes were put into ChatGPT‐4o and also given to two neurologists. ChatGPT answers to 20 FAQs were analyzed by three neurologists using the modified Discern score, the Global Quality score, and the Usefulness score. Text readability was assessed using the Flesch–Kincaid Reading Grade Level, the Flesch Reading Ease Score, and the Simple Measure of Gobbledygook.

**Results:**

The agreement between the neurologists and ChatGPT was fair. The  answers to the FAQs were of good quality, but unreliable and difficult to read.

**Conclusions:**

ChatGPT may be an additional valid tool for professional medical education. The quality of the answers to the FAQs is good, but the text is aimed primarily at highly educated individuals. The readability and reliability of the output would likely improve by providing the chatbot with specific prompts.

Recently, artificial intelligence (AI) has been applied to the healthcare field, being currently widely used in medical training to increase the accuracy of diagnoses, facilitate data‐driven decision‐making, and improve personalized patient management.^1^, ^2^ The most AI medical applications are typically based on Machine Learning (ML).^1^, ^2^ ML algorithms produce solutions based on patterns and schemas derived from previously provided data.^3^ One of the most recent subsets of ML, called Deep Learning (DL) techniques, falls within the so‐called generative AI, able of “reasoning,” analyzing, and generating content that resembles that generated by human intelligence.^3^ In the last few years AI has also been applied in various fields of neurology. In particular, neural network models have been developed for the collection, analysis, and interpretation of EEG data. Furthermore, AI technology‐based imaging has been implemented to enhance the detection of large vessel occlusions, and improve decision making in stroke thrombectomy.^2^ In movement disorders the potential of AI has been particularly focused on Parkinson's disease (PD) and has been exploited to improve deep brain stimulation settings, and develop wearable sensors and other motion capture systems.^2^

Chat Generative Pre‐trained Transformer (ChatGPT) is an AI language model based on DL techniques developed by OpenAI and designed to generate responses to text‐based inputs provided by users, closely miming human language patterns. The last version 4o has multimodal skills, accepting input and providing output not only as text, but also as images, audio, and video. ChatGPT is currently and widely applied to medical formation and diagnosis, and treatment recommendations. Recently, because ChatGPT is free, easy to use, and accessible, it has been increasingly consulted by patients and/or caregivers for a wide range of health issues, sometimes indiscriminately and uncontrolledly.

We aimed to evaluate the accuracy and reliability of ChatGPT's performance when consulted by healthcare professionals on specific PD‐related questions. Additionally, we assessed the quality and readability of ChatGPT outputs in relation to the most frequently searched PD topics online, to see whether this chatbot could be a useful resource for disseminating medical information.

## Methods

Based on medical training courses for neurologists, scientific sources and current guidelines, one author (ADR) developed 22 multiple choice quizzes with only one correct answer among four options that addressed the clinical and therapeutic features of PD (File S1). Two neurologists (AG, GR), with experience in movement disorders, also answered the same quizzes. ADR selected 20 FAQs about PD from the most popular patient information websites or those related to accredited scientific institutions (File S1). Furthermore, the same author relied on the most common questions received from PD patients in clinical practice. Both sets of questions were entered individually in Italian into ChatGPT (Version 4o, Open AI Inc. San Francisco, CA, USA) using the free interface available to consumers on September 29, 2025, in two distinct sessions, after clearing the computer's history. The initial response to each question was recorded without further research on the same topic or requests for clarification regarding the chatbot's feedback. We did not provide the chatbot with specific instructions, such as assigning specific roles or providing detailed context (eg, education level). The model was not explicitly set up to provide scientific sources, links, or citations of updated guidelines. All three neurologists (ADR; AG and GR blinded to the origin of the responses) critically and individually rated ChatGPT answers to the FAQs for clarity, educational quality, reliability and utility. The tools used were the following: modified Discern (mDiscern) score to assess the health information reliability^4^; the Global Quality score (GQS) for quality, flow of information and educational content^5^; Usefulness score (US).^6^ In the mDISCERN scale, each question is scored either 0 or 1 point, and a total score of 5 suggests better reliability, whereas a score of 0 demonstrates worse reliability.^4^ The GQS is rated in three categories, with scores of 1–2 indicating low quality, 3 intermediate quality, and 4–5 high quality.^5^ US ranges from 1 (lowest score) to 7 (highest score).^6^ Finally, using an online software, the readability of ChatGPT answers was assessed using the Flesch Kincaid Grade Level (FKGL), the Flesch Reading Ease Score (FRES), and the Simple Measure of Gobbledygook (SMOG) index. FKGL is a readability formula that provides a score corresponding to the education level required to understand a text, based on average sentence length and word complexity. The SMOG index is also a readability tool that estimates the years of education needed to understand a text. FRES evaluates the ease of understanding of a text by assigning a numerical score between 0 and 100; higher scores suggest simpler and more readable content.

### Statistical Analysis

Statistical analyses were conducted using SPSS software package, version 30.0 (SPSS Inc., Chicago, IL, USA). Continuous variables were reported as mean ± SD, whereas discrete data were indicated as median and range. The inter‐rater concordance about the responses to medical quizzes (“right” or “wrong”) was assessed using Fleiss Kappa coefficient. The inter‐rater reliability about the assessment of quality, reliability and utility of ChatGPT outputs was evaluated using Kendall's coefficient. Both coefficients range between 0 (no agreement) to 1 (perfect agreement).

## Results

ChatGPT found the right answer among the four options provided for 20 quizzes. The wrong ChatGPT answers concerned the exclusion criteria for PD diagnosis, according to current criteria,^7^ and risk factors for the development of motor fluctuations. The inter‐rater agreement about the medical quizzes answers was fair (Fleiss kappa value: 0.302; *P* = 0.014).

Table 1 shows the median values and ranges of the mDiscern score, GQS, and US obtained by each rater. Overall, at mDiscern scale, all ChatGPT outputs to PD FAQs were considered poorly reliable by all raters, with a good inter‐rater concordance. Conversely, based on GQS, the information provided was considered to be mostly of good or excellent quality and relevant. Finally, using US, ChatGPT answers were considered very or extremely useful for patients, regardless of the topic covered. The inter‐rater concordance was good for mDiscern score (Kendall's coefficient = 0.741; *P* < 0.001) and GQS (0.648; *P* < 0.001), and moderate for US (0.543; *P* < 0.001).

**TABLE 1:** Scores of reliability, quality and usefulness assigned by the three rater neurologists

Question	mDiscern	GQS	US
1	3^a^ (3–4)°	4 (4–5)	6 (4–7)
2	2 (2–3)	4 (3–5)	6 (3–6)
3	4 (3–4)	3 (3–4)	5 (4–5)
4	3	5 (3–5)	6 (2–6)
5	2 (2–3)	4 (3–5)	5 (2–7)
6	3 (2–3)	4 (4–5)	3 (3–7)
7	3 (2–3)	4 (2–5)	4 (3–7)
8	3 (2–3)	5 (4–5)	6 (6–7)
9	2 (2–3)	4 (4–5)	7 (5–7)
10	2 (2–3)	5	7 (6–7)
11	2 (2–3)	5 (4–5)	7 (6–7)
12	2 (2–3)	3 (2–5)	5 (2–7)
13	2 (2–3)	5	6 (4–6)
14	2 (2–3)	4 (3–5)	6 (3–7)
15	2 (2–3)	4 (3–5)	6 (3–7)
16	2 (1–3)	3 (3–5)	5 (3–7)
17	3	4 (4–5)	5 (5–7)
18	2 (2–3)	4 (4–5)	6 (3–7)
19	2 (2–3)	5 (4–5)	6 (3–7)
20	2 (2–3)	5 (4–5)	7 (5–7)
Kendall's coefficient	0.741	0.648	0.543

Abbreviations: mDiscern, Discern modified score; GQS, Global Quality Score; US, Usefulness Score; °, range.

^a^ Median.

The FKGL, FRES and SMOG tools suggested very poor readability of the ChatGPT responses, as the text was mostly extremely difficult to read and suitable for people with high level of education (Table 2).

**TABLE 2:** Reading grade levels of the answers to the FAQs provided by ChatGPT‐4o

	FKGL	SMOG	Education	FRES	Readability level
Question 1	14.79	18.75	Graduate	3.17	Extremely difficult
Question 2	12.71	15.77	Graduate	18.30	Very difficult
Question 3	15.00	23.99	Graduate	13.15	Very difficult
Question 3	11.04	15.25	Graduate	39.61	Difficult
Question 4	14.26	18.31	Graduate	23.75	Very difficult
Question 5	16.25	18.49	Graduate	1.62	Extremely difficult
Question 6	14.60	19.17	Graduate	14.17	Very difficult
Question 7	13.94	17.12	Graduate	21.94	Very difficult
Question 9	19.57	21.54	Graduate	0.0	Extremely difficult
Question 10	13.18	16.67	Graduate	19.99	Very difficult
Question 11	18.12	18.31	Graduate	0	Extremely difficult
Question 12	14.54	17.95	Graduate	9.09	Extremely difficult
Question 13	17.97	26.07	Graduate	0	Extremely difficult
Question 14	14.82	17.03	Graduate	9.78	Extremely difficult
Question 15	15.04	18.14	Graduate	13.32	Very difficult
Question 16	16.03	19.29	Graduate	13.30	Very difficult
Question 17	16.72	17.41	Graduate	3.07	Extremely difficult
Question 18	18.17	19.08	Graduate	0	Extremely difficult
Question 19	15.60	19.08	Graduate	5.42	Extremely difficult
Question 20	15.76	19.08	Graduate	5.75	Extremely difficult
Mean ± SD	15.0 **±** 2.0^a^	18.8 **±** 2.5^a^	‐	10.8 **±** 10.3*	‐

Abbreviations: FKGL, Flesch Kincaid Grade Level; FRES, Flesch Reading Ease Score; SMOG, Simple Measure of Gobbledygook index.

^a^ Mean ± SD.

## Discussion

ChatGPT evolving ability to produce accurate and detailed information is encouraging many healthcare professionals to use the chatbot to resolve medical issues and obtain support or advice on diagnostic and treatment options. Furthermore, due to free availability and ease of access, it is recently becoming an attractive source of healthcare information for laypeople.

To date, there is little data available in the literature on the consistency and appropriateness of online AI responses to questions regarding PD. A previous study explored the readability, reliability, and quality of ChatGPT performance regarding exercise recommendations for PD patients.^8^ Although ChatGPT output was correct and applicable, the authors found the text of the responses to be poorly readable. Similar results were obtained from an older study examining the readability of consumer‐oriented PD web page content using the FKGL and SMOG formulas.^9^

Here we assessed whether this chatbot may be useful in professional medical training and education, and represent a free, useful and reliable means of healthcare dissemination about PD for patients and/or caregivers.

In our study, ChatGPT performed comparably to neurologists in answering quizzes on clinical, diagnostic, and therapeutic features of PD, showing a fair level of concordance. ChatGPT performed worse in response to questions related to the latest current diagnostic criteria and risk factors for the development of motor fluctuations. The chatbot automatically provided reliable sources only in relation to one of the 20 FAQs, and this could have affected the reliability rating assessed using mDiscern score. This finding could suggest a potential negative impact on diagnostic and treatment decisions by online consultation, especially if inaccurate information is not critically analyzed and properly verified by healthcare professionals using valid scientific sources, current recommendations and guidelines. However, the chatbot was not explicitly or previously instructed to list scientific sources or links, or to base its outputs on current guidelines. Defining these exact prompts would likely have improved the reliability assessment.

All three neurologists considered the information provided to be of good quality and useful for patients. In contrast, readability scores were consistently low for all ChatGPT responses, because a college degree was required for understanding them. The discrepancy between the positive ratings by the physicians and the poor readability scores is very likely explained by the high level of education of the raters.

The most difficult answers to read were about typical PD symptoms, diagnostic approaches, association with dementia, best treatment for motor and non‐motor symptoms, constipation management, focused ultrasound applicability, search on stem cell transplantation, and undergoing anesthesia. As the average education level of the general population is lower than that needed to read the chatbot's outputs, the complexity of the sentences and use of scientific terms could impact ChatGPT effectiveness and actual applicability. The poor readability could be due to the lack of additional information provided to the model, such as the education level of the users, which generally leads to more personalized chatbot's output. However, our primary aim was simply to “simulate” a typical consultation by an individual with an average education level, who uses a chatbot in a “basic” way and is unable to train the model to respond to specific, personalized, and clear requests, or to use it interactively.

Similar issues with poor readability and reliability of content have been found in previous research aimed at assessing whether ChatGPT could be a valid information tool for other diseases such as fibromyalgia, orthopedic conditions and spinal cord injuries.^10^ These findings highlight the importance of refining specific and detailed prompts for future research on the topic, in order to ensure more accurate and usable results from ChatGPT and an objective evaluation of its performance.

Our study has other limitations. We analyzed ChatGPT responses to only 20 FAQs, which may not fully represent the wide range of PD topics searched online. The outputs were reviewed only by neurologists and not by PD patients or laypeople, and this could represent a significant bias due to the former's own scientific knowledge and clinical experience. Furthermore, we did not compare ChatGPT with other chatbot systems, and finally, we did not assess whether entering the same questions in English would have resulted in improved readability and reliability.

In conclusion, ChatGPT performance on knowledge of PD topics is comparable to that of neurologists. This finding suggests that it could be an additional tool for medical training and to support clinical practice, while taking into account its limitations and weaknesses.

AI systems such as ChatGPT‐4o have proven to be promising tools in improving patient education based on the quality and accuracy of information, but the terms currently used seem to be predominantly suitable for subjects with high cultural and education level. Therefore, the poor readability of ChatGPT outputs could limit its applicability and usefulness for low‐health‐literacy users, unless the model is provided with exact prompts, such as assigning roles and specific demographics. Consequently, indiscriminate, “basic” and unfiltered use of ChatGPT could be risky, especially for people who tend to self‐medicate and are unfamiliar with chatbots.

Overall, it would be helpful to inform lay users that any medical or health‐related research requires the entry of detailed input before entering questions, such as education level, in order to obtain safe, reliable, and personalized results.

## Authors Roles

(1) Research project: A. Conception, B. Organization, C. Execution; (2) Statistical Analysis: A. Design, B. Execution, C. Review and Critique; (3) Manuscript Preparation: A. Writing of the first draft, B. Review and Critique.

G.R.: 1B, 1C, 2C, 3B

R.C.: 1B, 1C, 2C, 3B

M.R.C.: 1C, 2C, 3B

A.G.: 1B, 1C, 2C, 3B

A.D.R.: 1A; 1B, 1C, 2A, 2B, 2C, 3A, 3C

## Disclosures

**Ethical Compliance Statement:** Due to the nature of the research, ethics committee approval was not required. Informed patient consent was not necessary for this work. We confirm that we have read the Journal's position on issues involved in ethical publication and affirm that this work is consistent with those guidelines.

## Financial Disclosures and Conflicts of Interest

Author disclosures are available in the Supporting Information.

## Supporting information


**File S1.** (A) Professional medical questions entered into ChatGPT‐4o.
(B) Frequently Asked Questions entered into ChatGPT‐4o.

**Data S1.** COI_disclosure.

## Data Availability

The data that support the findings of this study are available from the corresponding author upon reasonable request.
