# Supplementary material for: Quality and Readability of ChatGPT Performance on Parkinson's Disease
Source: Mov Disord Clin Pract. 2026 Jun 6:10.1002/mdc3.70700. Online ahead of print. doi: 10.1002/mdc3.70700 (PMC13338990; doi:10.1002/mdc3.70700)
Supplement: Supplementary file 1 — File S1. (A) Professional medical questions entered into ChatGPT‐4o. (B) Frequently Asked Questions entered into ChatGPT‐4o. [file MDC3-9999-0-s002.docx]

**A.**

| **Question** |  |
| --- | --- |
| **1** | What is one of the classic symptoms used to make a clinical diagnosis of Parkinson's disease in the early stages of the disease? |
| **2** | Which symptom can precede the clinical diagnosis? |
| **3** | What diagnostic approach can help confirm the diagnosis of Parkinson's disease? |
| **4** | According to the new diagnostic criteria for Parkinson's disease, which symptom would not exclude the condition in the differential diagnosis? |
| **5** | Which of the following is not a clinical feature associated with a worse prognosis in Parkinson's disease? |
| **6** | Which of the following is the most common symptom in Parkinson's disease at presentation? |
| **7** | Which of the following laboratory or imaging tests is necessary to confirm the diagnosis of Parkinson's disease in patients with typical progression? |
| **8** | Which statement is true regarding the management of Parkinson's disease? |
| **9** | Which statement is true regarding drug therapy for early Parkinson's disease? |
| **10** | Which factor is associated with a higher risk of motor fluctuations in Parkinson's Disease? |
| **11** | Which statement is true about the deep brain stimulation (DBS)? |
| **12** | Which of these statements about the symptoms of Parkinson's disease is correct? |
| **13** | Which of the following statements is true regarding cognitive decline in patients with Parkinson's disease? |
| **14** | Which of the following statements is true regarding punding and other impulse control disorders in Parkinson's disease? |
| **15** | Which of the following statements is true regarding fluctuations in non-motor symptoms in Parkinson's disease? |
| **16** | What is the most common type of levodopa-induced dyskinesia? |
| **17** | Which type of levodopa-induced dyskinesia typically occurs at the beginning and end of levodopa administration? |
| **18** | Which of the following is not considered a risk factor for the development of motor fluctuations in Parkinson's disease? |
| **19** | What could be one of the possible causes of reduced levodopa absorption in the gastrointestinal tract? |
| **20** | Which statement is true regarding motor fluctuations in Parkinson's disease? |
| **21** | Which statement is true about the "wearing off" phenomenon in Parkinson's Disease? |
| **22** | Which statement is true regarding subcutaneous infusion of foslevodopa/foscarbidopa in Parkinson's disease? |

**B.**

| **Question** |  |
| --- | --- |
| **1** | What are the symptoms of Parkinson's disease? |
| **2** | Is it Parkinson's disease even if there is no tremor? |
| **3** | How is Parkinson's disease diagnosed? |
| **4** | Is Parkinson's disease hereditary? |
| **5** | Does Parkinson's disease cause dementia? |
| **6** | Can I recover from Parkinson's disease? |
| **7** | Can Parkinson's disease be slowed? |
| **8** | Can I drive if I have Parkinson's disease? |
| **9** | What is the best treatment for Parkinson's disease? |
| **10** | What is recommended to eat if I have Parkinson's disease? |
| **11** | How can constipation be treated in Parkinson's disease? |
| **12** | Can Parkinson's disease cause difficulty swallowing? |
| **13** | What exercises are recommended for patients with Parkinson's disease? |
| **14** | Does deep brain stimulation cure Parkinson's disease? |
| **15** | Can I stop all Parkinson's disease medications after brain stimulation surgery? |
| **16** | Is ultrasound therapy useful in Parkinson's disease? |
| **17** | Can stem cell transplants cure Parkinson's disease? |
| **18** | Can I undergo anesthesia for surgery if I have Parkinson's disease? |
| **19** | Can I take anti-inflammatories if I have Parkinson's disease? |
| **20** | Can I take antibiotics if I have Parkinson's disease? |
